# Tissue Preservation and Access: Modern Innovation in Biobanking Moving Forwards a Personalized Treatment

**DOI:** 10.3390/jpm15050190

**Published:** 2025-05-07

**Authors:** Chiara Tessari, Saima Jalil Imran, Nukhba Akbar, Gino Gerosa

**Affiliations:** 1 Department of Cardiac, Thoracic, Vascular Sciences and Public Health, University of Padua, I-35128 Padua, Italy; saima.imran@unipd.it (S.J.I.); nukhba.akbar@sbkwu.edu.pk (N.A.); gino.gerosa@unipd.it (G.G.); 2 Dr. Zafar H. Zaidi Center for Proteomics, University of Karachi, Karachi 75270, Pakistan

**Keywords:** preservation, durability, rejection, bioengineering

## Abstract

Tissue substitution and graft transplantation are currently the best treatment options for patients suffering from severe heart diseases. However, the limited availability of donors and the restricted durability of tissues applied in cardiovascular treatments result in a constraint on applicability and a suboptimal therapeutic approach that is still not fully resolved. There are multiple ways to preserve heart tissue grafts, and the choice of method is solely dependent upon the nature and complexity of the tissue and the length of storage. The conventional cold storage method provides the base to nearly all of the preservation protocols for short- and long-term storage. Short-term storage methods frequently rely on designing preserving solutions to protect the graft against warm and cold ischemia at the temperature above freezing point. As ice-nucleation is the major notorious phenomenon during graft preservation, the modern era of research is focusing on developing ice-free preservation techniques, termed vitrification. However, despite the promising outcomes of vitrification, there are several recognized hurdles required to be overcome to build a biobank of heart grafts for an extended period of time. Besides tissue deterioration due to extreme cold temperature, there is another extreme phenomenon of tissue rejection mainly caused by the presence of cellular antigens. The modern approach of decellularization has the potential to minimize the chances of tissue rejection by removing the cells and providing a structural support and sustained biochemical signal via keeping the extracellular matrix of the graft intact. In conclusion, both nano-warming and decellularization are the leading approaches that have great potential to store the graft tissue in its optimal form via keeping its viability safe for a longer time and extending its applicability. This review article outlines a variety of approaches for the preservation and bioengineering of tissue to fulfill the need for the availability of on-shelf long-lasting grafts both in clinical and laboratory setups.

## 1. Introduction

The prevalence of heart diseases is continuously growing around the world regardless of advancements in the medical field [1]. The spectrum of treatments for cardiac disease spans from medical therapy to surgical interventions. Surgical options encompass strategies such as bypass grafts to circumvent damaged tissue or repairing/replacing tissue in conditions like valvular diseases. While tissues are typically harvested from the patient (autografts) for procedures such as bypass grafts or pericardial patches, there are instances where these tissues may be insufficient or unsuitable. Alternatively, human donor tissues (allografts) are utilized, but chronic shortages pose challenges. To address these limitations, tissues sourced from animals, such as bovine or porcine tissues (xenografts), are often employed [2]. Historically, the tissues obtained by these sources were preserved for a short period in a solution such as glutaraldehyde as it offers advantages in terms of availability and versatility but is limited in durability due to calcification processes and the immunological reaction known as host-versus-graft rejection, particularly problematic for younger patients. Despite the large portfolio of substitutes currently available, none fulfill the aims required for the “ideal substitutes”: mimic the characteristics of a normal native graft, be durable, have effective hemodynamics, be biocompatible, and often be always off-the-shelf.

In this scenario, tissue preservation becomes a critical consideration to ensure off-the-shelf availability.

As the challenges related to tissue graft preservation are inevitable and require thorough research and management protocols, this review mainly focuses on graft preservation methods while providing an insight into modern approaches to reduce the risks of injury by enhancing grafts’ tolerance and structural stability, as well as minimizing the chances of immune rejection for a long-lasting application independently from donor–recipient compatibility, thus making this a topic of significant clinical importance and innovation towards successful application.

A review of the literature has been performed using the following Keywords: tissue graft preservation, cryopreservation of tissues, surgical treatment of heart diseases, and short- and long-term tissue preservation protocol. We searched these terms on PubMed and Google scholar. Apart from the pioneering discoveries, all the literature was limited to the last 5 years.

## 2. Tissue Preservation Techniques

The main aim of tissue preservation is to ensure the sustainability of the microvascular, cellular, functional, and structural integrity of the graft prior to utilization. It also offers the on-shelf availability of the graft to avoid delays in treatments, as well as facilitate clinical practices and laboratory experimentations. Currently, there are a number of preservation methods under practice, including chemical fixation, combined physical and chemical approaches, cryopreservation, vitrification, decellularization followed by freeze-drying, and nano-technology-based approaches. The current review provides a critical overview of each method and comparative analysis to suggest the best possible approach in terms of stability, functionality, cost effectiveness, and personalized treatment.

### 2.1. Chemical Fixation

Chemical fixation is a common practice to preserve both allogeneic and xenogeneic tissues for a long time period to avoid tissue damage in an off-shelf setting. A vast number of chemicals were initially used for this purpose; however, glutaraldehyde is preferable due to its effective cross-linking, availability, cost-effectiveness, and efficiency. Over the past 50 years, glutaraldehyde has been favorably used to preserve and stabilize the tissue graft, especially for bioprosthetic valve substitutes. It improves graft stability by resisting the disruption of inter- and intramolecular proteins, reduces antigenicity, and maintains the sterility of the tissue during preservation [3]. Despite the advantages of using glutaraldehyde in clinical practices, many studies have mentioned the deterioration of tissue often characterized by calcification. This was mentioned by Pires et al., who noticed the location-based functional differences between membranous (smooth) and fibrosis (rough) sides of pericardium preserved in glutaraldehyde [4]. It has been recognized that residual glutaraldehyde-derived polymers serve as calcium-binding sites, promoting calcium phosphate deposits. In addition, phospholipids debris released from cell membranes, phosphates from free nucleic acids, and the mitochondria of dead cells act as calcium-binding sites [5]. Furthermore, calcific deposits mainly develop in areas where cusp flexion and stress are greater, such as the basal and commissural attachment points [6]. Several experiments have been designed to reduce the calcification tendency of glutaraldehyde-treated grafts, but this problem has remained. Although chemical fixation is a simple method which requires no special equipment and skills, there are significant risks, such as loss of tissue durability and regenerative capacity, calcification, and immune rejection. Due to the potential disadvantages of chemical fixation, the combined physical and chemical approaches were introduced to ensure the long-term storage of tissue grafts.

### 2.2. Combined Physical and Chemical Approaches for Graft Preservation

While the glutaraldehyde-based chemical preservation method is still under practice, the associated disadvantages lead to the development of alternative preservation strategies which primary focus on the use of physical parameters, particularly temperature control during graft preservation. The Arrhenius equation has helped researchers to generate this idea of graft preservation by understanding the mechanics of cellular processes, i.e., the rate of cellular processes slow down by 10% with a decrease in temperature of every 10 °C. For this purpose, two widely used techniques, i.e., static cold storage (SCS) and machine perfusion (MP), were developed that incorporate both physical and chemical factors to maintain graft viability.

#### 2.2.1. Static Cold Storage (SCS): Short-Term Graft Preservation

The static cold storage (SCS) method basically involves the immersion of grafts in a storage solution between the temperatures of 0 and 4 °C. The hypothermic condition not only maintains cell viability by decelerating the breakdown of vital compounds but also preserves energy by slowing down the cellular metabolism. The hypothermic condition surely preserves energy, but there are high risks of cell damage due to cell edema, calcium buildup, impaired Na^+^/K^+^ ATPase activity, cell acidosis due to ROS production, biomolecular damage, and the accumulation of toxic metabolites [7]. To overcome these challenges, the choice of storage solution is important as it maintains the structural and functional integrity of the membrane and prevents energy depletion, intracellular acidosis, and cell swelling of the graft. In certain situations, the use of colloids in storage solutions has also been tested to modify the SCS method to generate better outcomes [8,9]. Prior to implantation, the retrieval of the graft on body temperature is also a critical step followed by SCS. The rewarming of the graft may also cause reperfusion injuries due to temperature elevation and oxygen load [10]. Thus, this may result in delayed myocardial recovery, primary graft failure, and atherosclerosis. These issues impose time constraint on SCS: the longer the graft remains in SCS, the greater the potential for graft damage. Consequently, while this preservation method is widely adopted for whole heart transplantation, it is not commonly employed for cardiovascular tissue applications. Moreover, even though the SCS method is economical, user-friendly, and is ideal for the short-term storage of the heart, the associated risks have encouraged researchers to develop a dynamic approach of the machine perfusion method to maintain long-term graft viability by continuously providing optimal conditions.

#### 2.2.2. Machine Perfusion Method: Dynamic Approach for Graft Preservation

Machine perfusion is an advanced method involving graft perfusion with the controlled flow of oxygenated solution that not only maintains the microvascular structure of the graft but also provides a controlled environment to ensure the supply of nutrients as well as the removal of toxic metabolites. Hence, it significantly prevents irreversible ischemia to the graft and consequent cell death [11] for the treatment of IHDs and HFs, two types of machine perfusion systems that have been employed according to temperature variations, i.e., normothermic machine perfusion (NMP) and hypothermic machine perfusion (HMP) [12].

Normothermic machine perfusion (NMP) was initially used for the assessment of graft function prior to transplantation. It is implied to preserve the grafts by providing physiological temperature (35–38 °C) to maintain tissue viability [13]. The NMP approach has been used to preserve a vast variety of organs such as liver, kidneys, and hearts. The researchers believe that the NMP approach is superior to the static cold storage method in terms of preserving the hearts of dogs and pigs [14,15]. Clinical trials over a 2-year period have proved the advantage of NMP over statice cold storage in terms of a low rate of primary graft failure and recipient survival by studying 159 cases of orthotopic heart transplantation [16]. Currently, the Organ Care System™ (TransMedics, Andover, MA, USA) and Organ Assist^®^ device (Organ Assist, Groningen, The Netherlands) are commercially available devices, manufactured on the principle of NMP for the preservation, transport, and functional assessment of the heart, liver, lungs, and kidneys for clinical practices.

Hypothermic machine perfusion (HMP) is based on preserving the graft at temperatures below 10 °C for up to 48 h. Although NMP has better outcomes and is preferred in clinical setups, it was speculated that long-term preservation is more likely achievable by reducing cellular metabolism under hypothermic conditions. During the process of HMP, the graft is subjected to a cold storage solution enriched with oxygen, nutrients, hormones, and RBCs for the restoration of ATP level during storage [7,13]. There are a limited number of studies reported for using HMP in clinical setups; however, all those studies have reported positive results as compared to SCS, particularly for the preservation of the lungs and heart [16].

Although the machine perfusion approach has many advantages, these approaches are still not devoid of limitations. The very first drawback of the machine perfusion method is its high cost, which limits its application only for the preservation of the whole heart for transplantation. Other drawbacks are the need for technical experts for the configuration and implementation of the device and its specialized means of transportation. Furthermore, both SCS and MP methods only allow for the preservation of a graft for a short period of a few hours, and any further delay may result in a 70% early mortality risk [8].

### 2.3. Cryopreservation: Classical Approach

It is experimentally confirmed that tissue/organ drafts can be stored for a very long period at a very low temperature. For the first time in 1948, Smith and Parkes dramatically developed a technique currently known as “cryopreservation” by freezing spermatozoa at −70 °C in glycerol solution [17]. So far, a wide range of cells such as spermatozoa, lymphocytes, erythrocytes, endocrine cells, hematopoietic cells, and tissues have been reported to be preserved by cryopreservation. Cryopreservation is a classical approach implied by the nature and concentration of cryoprotective agents (such as glycerol), the temperature on which the cryoprotective agent is added, storage temperature, the rate of cooling/thawing, and the temperature on which the cryoprotective agent is removed. There are multiple factors required to be optimized to make the classical cryopreservation approach suitable for all kinds of tissue/organ grafts. These factors included the magnitude and cellular localization of ice-crystals, toxicity of cryoprotective agents, the effects of temperature, the magnitude of osmolality, and its effects on cell volume [18]. As these factors possess significant challenges to the idea of long-term graft preservation, many researchers have put their outmost efforts in to overcome these challenges and have modified classical cryopreservation in different ways (Table 1), such as supercooling, partial freezing, and isochoric preservation, as have been briefly discussed in a review [19]. All these techniques mainly focus on maintaining graft viability by controlling ice-crystal formation. These techniques rely on manipulating temperature, which consequently controls the tissue microenvironment, to inhibit tissue metabolism and prevent it from the detrimental effects of uncontrolled freezing. While supercooling and partial freezing control ice-crystal formation by utilizing cryoprotective agents (CPAs), the isochoric preservation method performs a similar function by maintaining constant volume. Despite the differences in their mechanism, all these techniques share the common purpose of graft preservation at sub-zero temperatures without compromising tissue integrity. Currently, directional freezing and vitrification offer several advantages over supercooling, partial freezing, and isochoric preservation methods due to their enhanced ability to control ice-crystal formation and superior graft preservation outcomes.

#### 2.3.1. Directional Freezing: Modified Cryopreservation Technique for Long-Term Graft Storage

Short preservation time causes the loss of donated tissue/organ grafts that contribute to failure to meet the global supply demands of grafts for transplantation. Directional freezing is an advanced approach used to preserve large organ and tissue grafts by precisely adjusting the temperature gradient to achieve uniform and accurate cooling throughout the graft [33]. It involves the long-term preservation of grafts via a gradual decrease in temperature, i.e., between −80 °C and −196 °C, without much altering of the physiological properties of the cell [34,35]. XDirectional freezing approach was first used to design a cryo-microscope [36]. The basic principle of directional freezing involves the slow linear motion of a graft with constant velocity through a temperature gradient by precisely controlling the rate of cooling, ice-crystal formation, and the latent heat transfer. Directional freezing has an advantage over cryopreservation in terms of avoiding the supercooling of the graft. A vast number of cell and tissue grafts have been successfully preserved by directional freezing, such as human and sheep ovaries, pig liver, and rat heart [27,28,29,30,31]. Although directional freezing has many advantages over static cold storage and machine perfusion methods, it has several shortcomings, such as the risks of uneven crystal formation and thermal stress that may cause damage to the graft. Additionally, the manufacturing of the whole setup for directional freezing is quite complicated and expensive, a skilled person is needed to perform it properly, and the whole process of directional freezing is time-consuming and there is high risk of graft degradation during the freezing process. To overcome these limitations, the researchers are currently exploring more advanced techniques of cryopreservation, such as vitrification.

#### 2.3.2. Vitrification: Modified Cryopreservation for Long-Term Graft Storage

Vitrification is a recently developed cryopreservation technique used for the successful preservation of human embryos and oocytes since 1998 [37,38]. Vitrification is considered an alternative technique to directional freezing as it involves the rapid cooling of the graft, contrary to slow uniform cooling [39]. It also prevents thermal stress of the graft, which leads to ischemic injury, as in case of directional freezing. So far, vitrification has been effectively used for the preservation of a vast number of tissues such as the kidneys, ovaries, vessels, valves, cornea, and cartilage [28]. Vitrification is based on a physical phenomenon in which a liquid is directly transformed into a solid glass-like state by extreme elevation in viscosity. It avoids the process of crystal formation and growth by maintaining the molecular arrangement of high-viscosity liquid in a glass-like state [28]. Additionally, vitrification is also implied using a high concentration of cryoprotectants that prevent ice-formation at low temperatures. Currently, nanotechnology is considered a synergistic approach that can prevent the formation of ice-crystals during the cooling/thawing cycle of the graft, as well as mitigating thermal stress.

## 3. Modern Perspectives for Long-Term Tissue Storage

### 3.1. Nanotechnology in Graft Preservation

The concept of nanotechnology was first introduced by physicist Richard Feynman in 1959 to manufacture things at the atomic or molecular level. Currently, nanotechnology also has great potential to overcome the limitations of currently available treatment options for heart diseases by having less toxicity and improved mechanical properties, biodegradability, and biocompatibility [40]. It is now considered a promising field to improve preservation, transportation, and preconditioning of the tissue to ensure successful implantation. The traditional static cold storage and cryopreservation techniques have the limitations of reduced cellular viability and altered tissue architecture due to ice-crystal formation during the freeze/thaw cycle. By using nano-materials, it has become possible to avoid the disastrous effects of the freeze/thaw cycle [19].

Nano-warming is a breakthrough that guarantees the preservation of grafts for the longest period of time while maintaining tissue viability and infrastructure.

#### Nano-Warming

**Figure 1:**
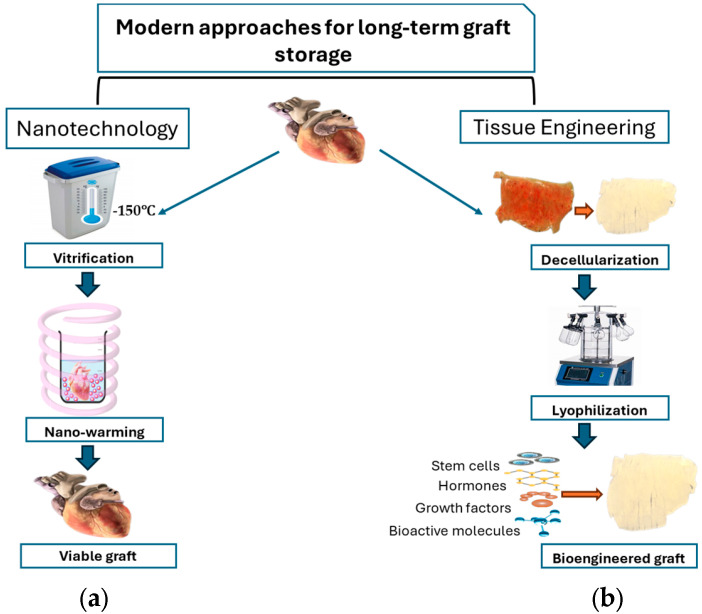
Schematic illustration of Nanotechnology (**a**) and Tissue culture technique (**b**).

Several methods for the rewarming of tissue grafts have been under practice, including convective warming, dielectric heating, laser heating, and microwave heating [41]. However, each method has its own limitations, such as thermal stress, uneven heating, and risks associated with laser penetration [41]. Nano-warming is a modern approach used for the rewarming of large vitrified grafts by applying energy at the nano-scale level [42]. For this purpose, magnetic iron oxide nanoparticles (mIONPs) are loaded into vitrified grafts and then exposed to a low-radiofrequency alternative magnetic field for warming (Figure 1a).

In contrast to high-frequency microwave warming, the low-radiofrequency magnetic field does not directly warm the grafts; rather, it facilitates the achievement of the desired heating rate through modifying the concentration of mIONPs and magnitude and frequency of the magnetic field [43]. Low-radiofrequency waves not only maintain tissue infrastructure but also ensure uniform heating throughout the tissue grafts.

So far, the nano-warming approach in combination with vitrification has been used to preserve either very thin tissue grafts like the heart valves and arteries or highly vascularized organs such as the kidneys and heart [44,45,46]. However, a recent study attempted to use nano-warming to preserve more complicated and large tissue grafts, i.e., large articular cartilage, and showed promising results for clinical applications of cryopreserved cartilage [47].

Despite the promising application of nano-warming in graft preservation, there are certain limitations, including the (1) stability of nanoparticles in the strong ionic environment of CPAs and (2) the uniform dispersal of nanoparticles within the graft, which require further research.

Vitrification combined with the nano-warming technique may bring about remarkable improvements for the ideal preservation of lager grafts in order to fulfill a patient-targeted treatment. It still needs several optimizations, e.g., exploring more suitable vitrification solutions, machine profusion protocols, efficient CPAs with minimum toxicity, and suitable nanoparticles alongside deep molecular understandings.

For the ideal preservation of larger grafts to attain the intact structural integrity, the decellularization-based tissue engineering method has recently come under profound consideration.

### 3.2. Tissue Engineering: A Revolutionary Approach in Graft Preservation

Regenerative medicine is a branch of medical sciences that aims to repair damage to the tissue/organ. It encompasses a vast number of techniques, including tissue engineering. The basic principle of tissue engineering comprises preparing tissue grafts with boosted biological activity and biocompatibility by exogenously fabricating basic biological entities, i.e., cells, biomaterials, and bioactive molecules [48,49]. The choice of biomaterials is based on their aptitude to support host cells and bioactive molecules, as well as provide structural scaffolds that mimic the mechanical properties of native tissue [50]. Primarily, tissue engineering approaches were relied on in the application of biomaterials from animal plants and synthetic origins repopulated with the cell (Figure 1b), which are further tested for tissue coherent contraction and other in vivo physiological activities after implantation. However, this traditional approach has resulted in some challenges, including immune rejection, poor cell migration, the biodegradation of scaffolds, poor tissue coherency, and vascularization [49]. Immune rejection is one of the most challenging factors in graft transplantation that has led scientists towards the decellularized extracellular matrix-based (dECM) approach. Decellularization of the graft removes the residual presence of xenoantigens, such as alfa-Gal, Neu5Gc, and others, while preserving the extracellular matrix to enhance biocompatibility [48]. There are multiple decellularized tissue grafts that are currently in clinical practice, including decellularized cardiac patches, cardiac valves, pericardium, and blood vessels. The dECM-based approach mainly focuses on developing a biocompatible 3D scaffold material with preserved geometry and vascular structure by the process of decellularization [51]. Most of the protocols of the decellularization of cardiac-related tissues are employed by various physical, chemical, and enzymatic methods. Physical methods include freezing, mechanical force, agitation, vacuum-assisted decellularization, and hydrostatic pressure. Chemical methods involve the use of EDTA, triton-X, SDS, hypotonic and hypertonic buffers, and acid–base solutions, while the enzymatic method involves the use of enzymes like pepsin, trypsin, endo, and exonucleases. The choice of decellularization method is based on the type of tissue and the purpose of the experimentation [52].

After decellularization, it is substantial to sterilize the tissue to further purify it from the residues of genetic material to reduce the risk of immunogenicity. The techniques used commonly for sterilization purposes include gamma and electron radiation, ethylene oxide (ETO), and super critical carbon dioxide (scCO_2_), with the occasional use of antibiotics during the decellularization process. So far, the use of scCO_2_ is considered to be more advantageous over other methods for thick tissue sterilization due to its low toxicity, low viscosity, and high penetration power. Thus, it causes less damage to the structural and mechanical properties of the grafts [53,54]. After decellularization and sterilization, the tissue can be preserved using short- and long-term methods, including cryopreservation, freeze-drying, and the −20 °C/−80 °C storage condition, along with the utilization of antibiotics. Likewise, cryopreservation is another way to store the grafts for a long term by using 10% DMSO, along with slow or snap rate freezing with liquid nitrogen [55]. Freeze-drying or lyophilization is so far the best method for long-term storage. The low pressure intends to speed up the freezing process, followed by a rise in temperature that further breaks the bond between water and the decellularized graft, thus causing further drying of the graft. This method is cost-effective, needs no toxic protective agents or chemicals, and eliminates the need for ultra-low-temperature storage; thus, it is considered a preferable method for graft preservation [20,56].

These scaffolds then can be repopulated. Regarding repopulation methods, two main approaches are pursued: in vitro tissue engineering and in vivo tissue-guided regeneration. In vitro tissue engineering represents the conventional concept, in which scaffolds are seeded with cells prior to implantation. Cells are added to the scaffolds inside a bioreactor, in which the construct is subjected to the necessary biochemical and mechanical stimuli. Although a fascinating concept, in vitro tissue engineering for heart valve substitute creation is far from clinical application. The first problem is the difficulty obtaining a mature heart valve from an artificial starting matrix, with proper biomechanical properties. Another problem is the risk of contamination during the in vitro tissue culture phase. Consequently, heart valve regenerative medicine shifts the paradigm towards the alternative concept of in vivo tissue-guided regeneration. The latter utilizes the natural regenerative potential of the human body for the repopulation of an implanted heart valve scaffold, avoiding cell and tissue culture steps [57]. The scaffold intended for tissue-guided regeneration may also undergo specific modifications and surface engineering to prepare for in vivo repopulation. For example, aptamers combined with star-polyethylene glycol coating have been investigated as a promising technique [58]. These methods have the potential to become viable tools for addressing the challenges of limited grafts from humans, rejection, and limited durability, thereby enabling personalized treatment approaches.

Tissue engineered decellularized cardiac valves are now commercially available, and their transplantation has demonstrated promising results. Compared to standard tissue conduits, implanted decellularized cardiac valves have exhibited less complexities and lower reoperation rates, as systemically reviewed by Waqanivavalagi [59]. The main aim of using decellularized cardiac valve was to minimize the risk of immune reactions. However, recent reports have elucidated the immune responses followed by decellularized valve transplantation, thus raising significant concerns [60,61]. Several strategies can help to resolve this concern, including reseeding decellularized conduits with host stem cells, coating the valves with chemicals such as glycerol to mask the surface antigens, and employing genetic engineering to eliminate antigens.

## 4. Conclusions

Although cryopreservation is a standard protocol used in clinical setups for the implantation of small tissue/organ grafts, for the larger tissues/organs, this method is not preferred due to the unrecoverable damage it may cause to their highly delicate structures. Additionally, cryopreservation allows for the preservation of graft tissue for a short span of a few days. The researchers have high hopes from the vitrification and nano-warming technique that it may bring about remarkable improvements for the ideal preservation of larger grafts and for longer time span, but it still needs a lot of optimizations, e.g., exploring more suitable vitrification solutions, efficient CPAs with minimum toxicity, and suitable nanoparticles alongside deep molecular understandings. The field of regenerative medicine is a novel approach for the ideal preservation of larger grafts by leveraging biological and engineering principles to improve graft availability and sustainability. By incorporating stem cells, bioactive molecules, growth factors, and hormones, the regenerative practices can not only maintain graft integrity but also promote tissue healing and the regeneration of injured cardiac tissue. Decellularization is considered to be one of the best approaches in regenerative medicine to attain the intact structural integrity of the graft while minimizing the risk of immune rejection and ensuring on-shelf availability for routine laboratory and clinical practices. However, it is very important to understand that both nano-warming and decellularization do not work independently but rather advanced approaches that complement and improve existing cryopreservation methods are needed.

A key area for future research and development is the concept of “depersonalizing” grafts to increase their viability among recipients with different immunogenicity profiles. This approach aims to modify grafts in ways that reduce their antigenic properties, potentially leading to decreased rejection rates and expanded donor pools. Strategies for depersonalizing may include removing donor-specific antigens, modifying surface proteins, or applying immunomodulatory coating. By focusing on this area, researchers may be able to develop more universally compatible grafts, bringing us closer to the ideal of “off-the-shelf” tissue and organ replacement. This line of research holds immense promise for overcoming the current limitations in graft preservation and compatibility, potentially revolutionizing the field of cardiovascular medicine.

## Figures and Tables

**Table 1:** Methods of cryopreservation in graft preservation.

Name of Method	Storage Temperature	Storage Time	Storage Phase	Conditions	Reported Tissue Grafts	Advantages	Disadvantages	References
Supercooling	−4 °C to −8 °C	2–6 days	Liquid	Cryoprotective agents (CPAs)	Liver and heart	Reduce ice-crystal formation. Allow homogenous distribution of CPAs throughout the graft. Minimize air–liquid interface to minimize ice-nucleation.	Short-term storage. CPAs-induced cytotoxicity.	[20,21,22]
Partial freezing	−10 °C to −15 °C	~5 days	Partially frozen state	Cryoprotecting agents (CPAs) and ice-nucleators	Liver	Maintain graft viability. Reduce ice-crystal formation.	Short-term storage. CPAs-induced cytotoxicity.	[23,24]
Isochoric preservation	~−8 °C	~2 days	Liquid non-frozen state	Constant volume	Liver and heart	Controlled ice-nucleation.	Short storage time. Complex setup. Risk of uncontrolled tissue freezing.	[25,26]
Directional freezing	−80 °C to −196 °C	For weeks	Partially frozen state	Progressive decrease in temperature	Ovaries, liver, and heart	Maintain tissue viability by avoiding supercooling.	Risk of un-even crystal formation. Complicated and expensive setup.	[27,28,29,30,31]
Vitrification and nano-warming	−80 °C to −150 °C	13 years	Glassy non-crystalline state	Combined use of CPAs with highly regulated rapid cooling system.	Embryos, Cardiac valves, arteries, and myocardia	Maintain cell viability at extremely low temperature. Prevent ice-crystal formation. Long-term storage.	CPAs-induced cytotoxicity.	[26,32]

## Data Availability

No new data were created or analyzed in this study.
